# Bacillary Angiomatosis Mimicking Pyogenic Granuloma

**DOI:** 10.7759/cureus.36844

**Published:** 2023-03-29

**Authors:** Ramesh Kumar A, Nitya K, Raj Kumar Krishnan, Snehesh Dinesh, Ramya R

**Affiliations:** 1 Oral and Maxillofacial Pathology, Sri Ramaswamy Memorial (SRM) Dental College, Chennai, IND; 2 Oral Biology, Saveetha Dental College and Hospitals, Chennai, IND; 3 Oral Surgery, Sri Ramaswamy Memorial (SRM) Dental College, Chennai, IND

**Keywords:** brown and brenn, warthin-starry, endothelium, pyogenic granuloma, gingiva, mandible, diabetes, bartonella, bacillary angiomatosis

## Abstract

Bacillary angiomatosis (BA) is an angioproliferative disease caused by *Bartonella* species. It manifests as nodules or papules in immunocompromised patients. Oral lesions are very rare, unlike cutaneous lesions, and histopathology plays a vital role in distinguishing these lesions from other similar ones. Treatment mainly comprises macrolides erythromycin, clarithromycin, or doxycycline.

## Introduction

Bacillary or epithelioid angiomatosis is a rare disease characterized by neovascular proliferation in the skin or internal organs that manifests as tumorlike masses caused by *Bartonella henselae* or *Bartonella quintana* infection. Previously, bacillary angiomatosis (BA) was thought to affect only HIV patients, but it is now known to affect people who are in non-HIV immunocompromised states and immunocompetent persons [1]. Most commonly, the disease affects the skin, but the involvement of the respiratory tract, bone, lymph nodes, and gastrointestinal tract has also been reported [2,3]. Oral involvement is rare, yet dental practitioners should be aware and carry out proper histopathological analysis for confirmatory diagnosis. In this paper, we report a case of bacillary angiomatosis mimicking pyogenic granuloma in the mandibular gingiva of a diabetic patient.

## Case presentation

A 46-year-old male patient reported a chief complaint of reddish growth in the lower front tooth region for the past eight months. Initially, the growth was small, and the patient only noticed the enlargement of the lesion in the last two months. The patient had a previous history of growth in the same region thrice that had resolved itself. The patient was found to be diabetic and has been put under medication for the past three months. Routine hematological investigations revealed leukocytosis, and fasting and postprandial blood glucose levels were 180 mg/dL and 230 mg/dL, respectively.

**Figure 1:**
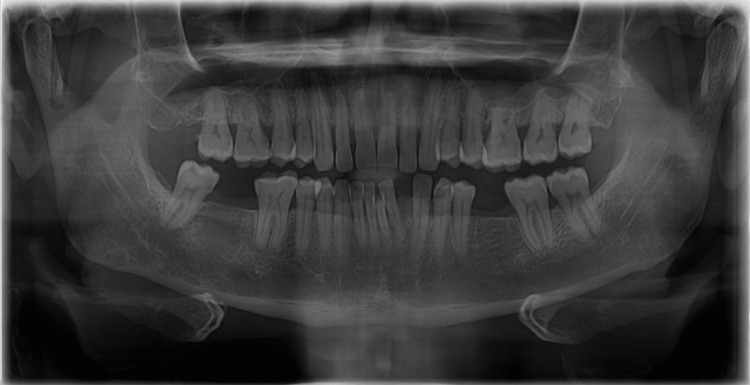
Orthopantomogram showing generalized chronic periodontitis and drifting of 32

On inspection, a solitary growth of 2 cm × 1 cm was seen extending from the mesial aspect 41-44 inferiorly to the anterior vestibule. The growth appears to be shiny and erythematous on the labial surface with normal color adjacent mucosa on the occlusal surface of the lesion. Bleeding is noted without pus discharge. On palpation, the growth was soft to fibrous in consistency, smooth, non-tender, and non-fluctuant, and bleeding was present without pus discharge. Submandibular/regional lymph nodes were palpable. The radiographic findings (orthopantomogram) revealed the presence of teeth from 16 to 28 and from 38 to 48. Complete loss of coronal and radicular structure is seen in 47 and 36. Generalized bone loss is seen until the middle third of the root with drifting of 32 (mesially) due to pathological migration, suggestive of chronic periodontitis (Figure 1). The maxillary sinus, condyle, coronoid notch, body, angle, and ramus of the mandible appears to be normal.

**Figure 2:**
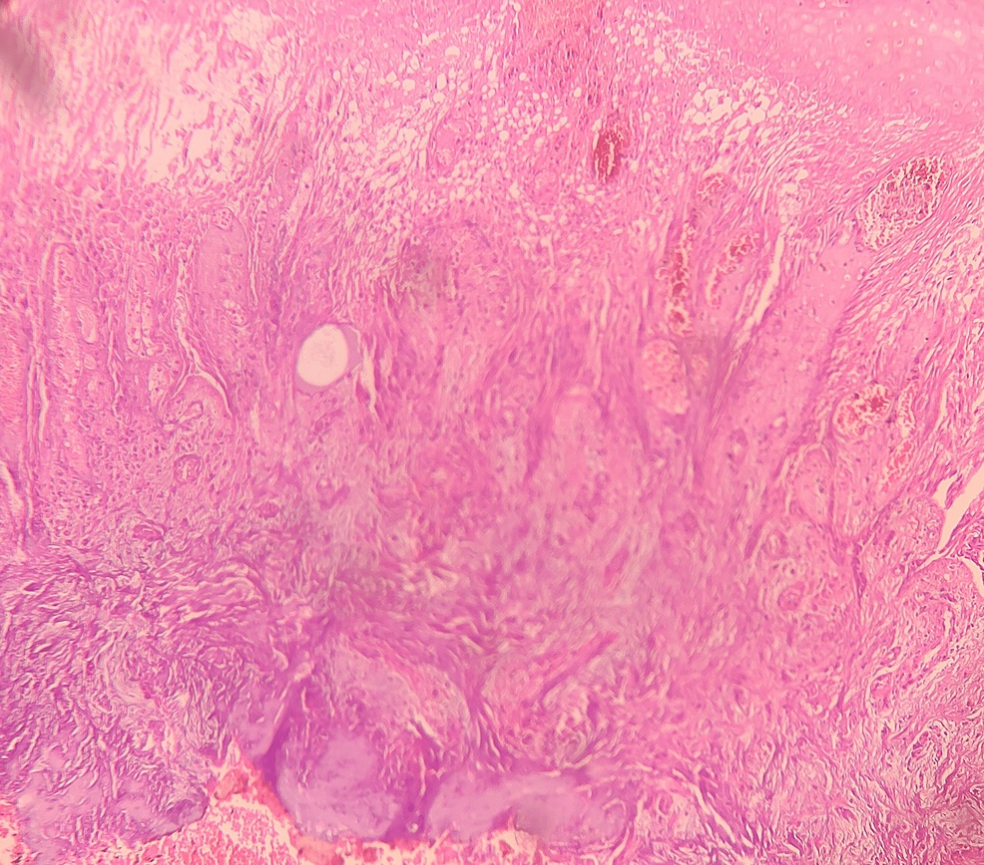
Hematoxylin and eosin-stained tissue section showing hyperplastic stratified squamous epithelium with intercellular edema

**Figure 3:**
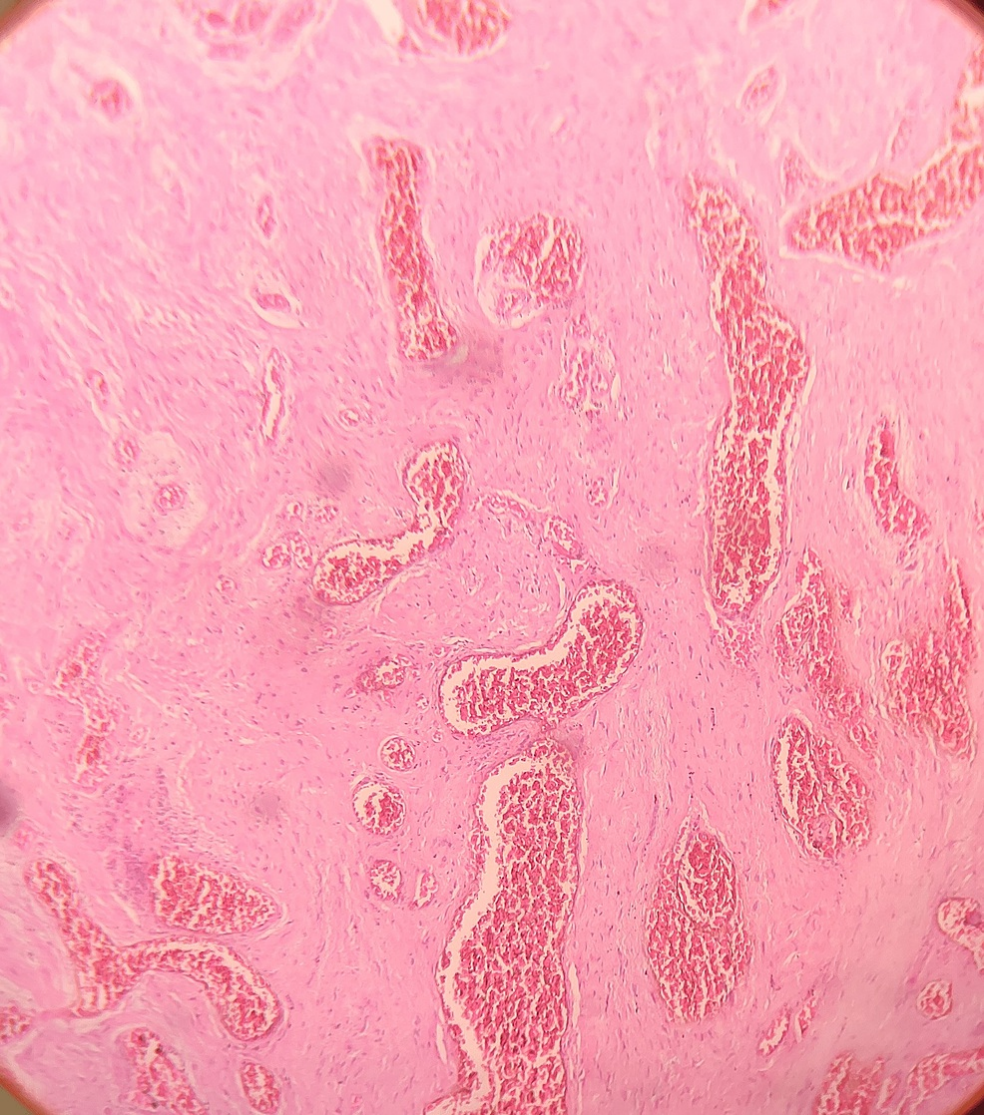
Hematoxylin and eosin-stained tissue section showing connective tissue exhibiting lobular proliferation of capillaries lined by prominent endothelial cells

**Figure 4:**
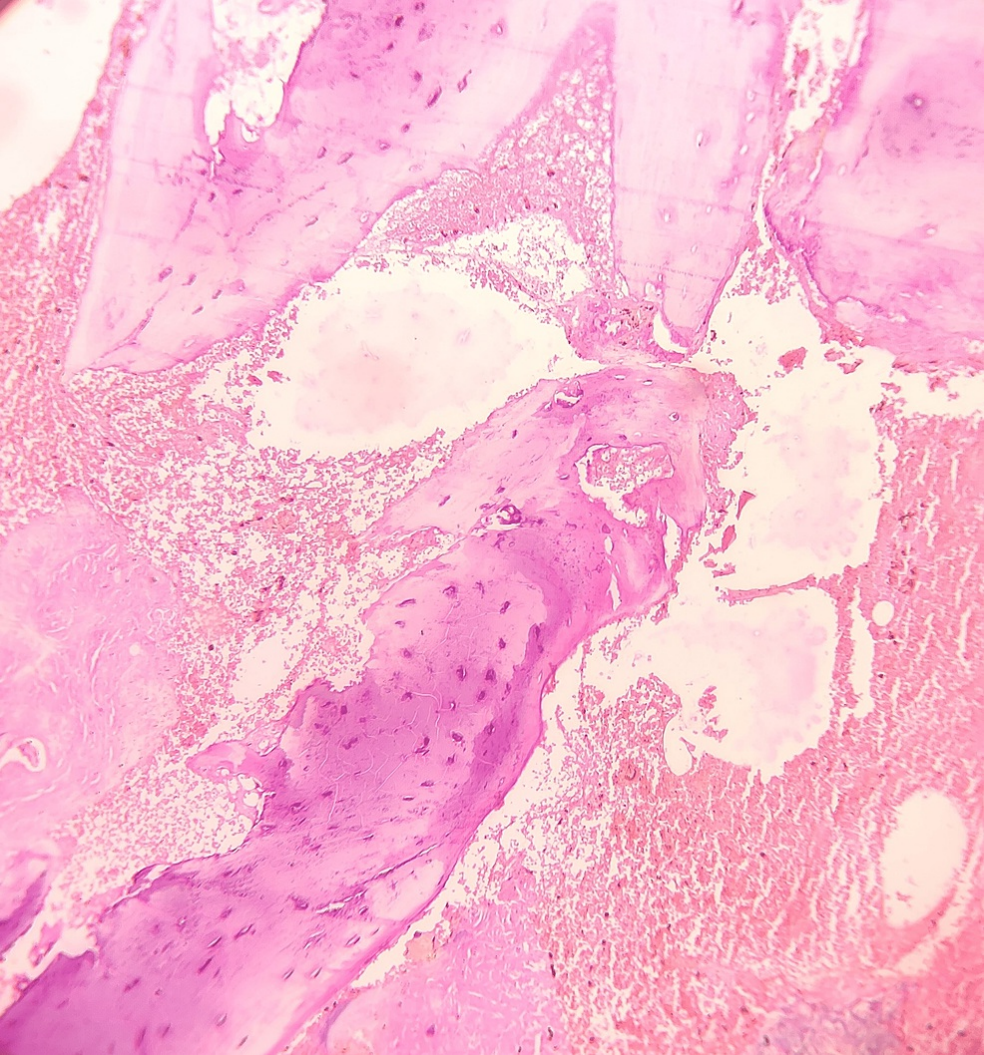
Hematoxylin and eosin-stained tissue section showing underlying alveolar bone

Based on the history and clinical findings, a provisional diagnosis of pyogenic granuloma was given. Under local anesthesia, an incisional biopsy was done, and the tissue was submitted for histopathological evaluation. Hematoxylin and eosin-stained tissue section revealed a hyperplastic stratified squamous epithelium. The epithelium seems to exhibit prominent intercellular edema. The underlying connective tissue exhibited lobular proliferation of capillaries lined by prominent endothelial cells. Leukocytoclastic debris and granular cytoplasmic material representing bacteria were noted. Inflammatory cells such as neutrophils, lymphocytes, and histiocytes were also present (Figures 2-4). The histopathological features are suggestive of bacillary angiomatosis. To confirm the presence of bacterial colonies, special stains such as the Warthin-Starry stain and the Brown and Brenn stain were carried out.

**Figure 5:**
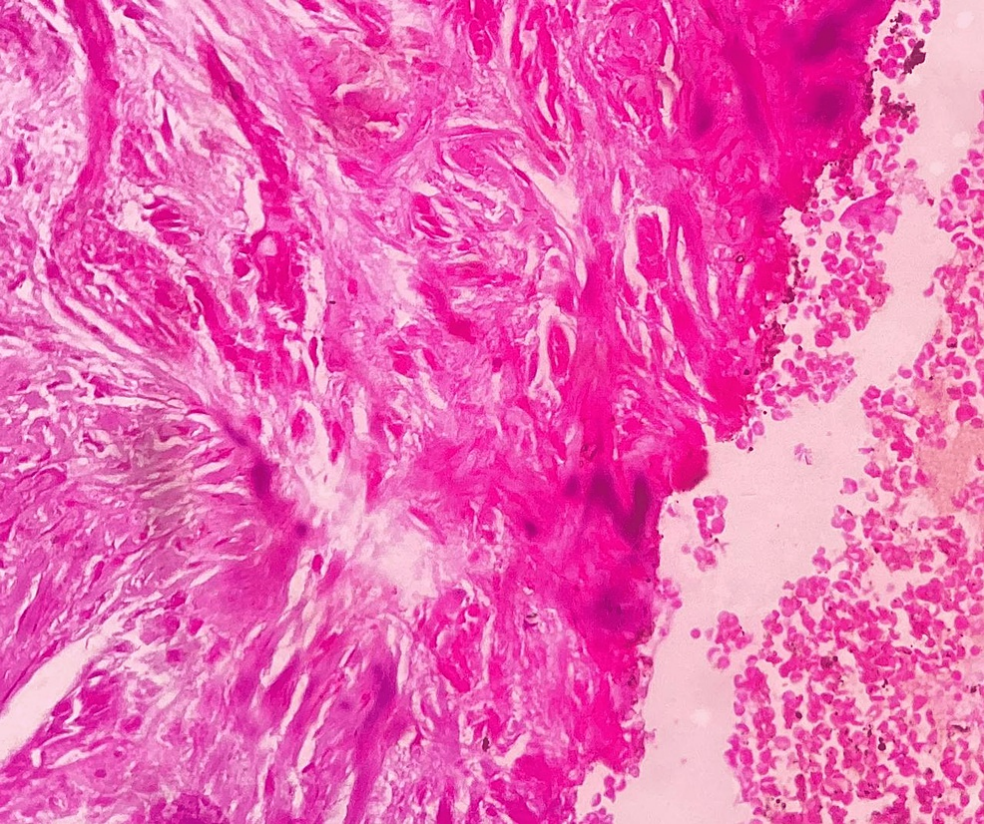
Brown and Brenn modified gram stain showing superficial microbial colonies (resembling gram-negative rods)

The Warthin-Starry stain showed the presence of black rod-shaped bacilli arranged in clusters. The modified Brown and Brenn stain showed superficial microbial colonies (Figure 5). IgG and IgM titer results were also found to be positive. Ciprofloxacin and ibuprofen were advised, and the patient is recalled after two months. The patient was advised to reduce blood sugar levels by following proper medication and diet.

## Discussion

Bacillary angiomatosis (BA) is a vasoproliferative lesion first described by Stoler in 1983. BA is caused mainly by *Bartonella henselae* or *Bartonella quintana*, mainly affecting immunocompromised individuals and also patients with advanced HIV diseases [4]. The typical cutaneous lesions of BA appear as single or multiple bright red to deep purple dome-shaped papules or nodules [5]. Cutaneous BA lesion’s clinical differential diagnosis may include Kaposi sarcoma (KS), pyogenic granuloma, cherry angioma, dermatofibroma, hemangioma, and mycobacterial infection such as tuberculosis, coccidioidomycosis, cryptococcosis, and histoplasmosis. BA lesions most commonly involve the skin, but the involvement of the respiratory tract, bone, lymph nodes, gut, and brain are also reported. With trauma, the lesions may bleed profusely and be singular or multiple in number, but they rarely appear disseminated [6,7]. Oral cavity involvement is very rare, and only a few cases have been reported (oral mucosa, hard palate, lips, and alveolar bone); its appearance is almost similar to pyogenic granuloma clinically [8,9].

*Bartonella henselae* is a zoonotic pathogen and was initially known as *Rochalimæa henselae* and is most commonly linked with the immune status of an individual. It is usually a self-limiting infection characterized by lymphadenopathy, fever, myalgia, and fatigue. *Bartonella henselae* associated with meningoencephalitis, endocarditis, ophthalmic involvement, bacteremia, and neurological disorders are also reported in immunocompromised individuals [10,11].

*Bartonella henselae* are small, pleomorphic, fastidious, facultative, gram-negative intracellular bacilli causing granulomatous and suppurative infections. Bacillary angiomatosis can occur in non-HIV patients such as transplant recipients, people with chronic hepatitis B, people with leukemia, and people on chemotherapy, especially when CD4 T-helper cell counts are suppressed. Bacillary angiomatosis may appear as a pyogenic granuloma at the site of a burn or a cat scratch in immunocompetent patients [12].

People with diabetes are more likely to get affected by bacterial infections (mainly gram-negative bacteria, as seen in the present case). Infections such as pertussis, cat scratch disease, and fungal-like candida are more prevalent. The clinical manifestations of *Bartonella henselae* infection vary in patients with immunocompromised status. So, a differential diagnosis of malignant neoplasms such as Kaposi sarcoma or angiosarcoma/benign conditions such as pyogenic granuloma and angiolymphoid hyperplasia with eosinophilia should be considered [13].

After initial inoculation, *Bartonella* could be rapidly cleared from the blood, which was thought to be due to *Bartonella* infection of the so-called primary niche outside of circulating blood, which included potentially endothelial cells, erythrocytic precursors, liver, and possibly other cell types or organs. Between two and five days after infection, *Bartonella* can be discovered in the bloodstream. Following that, erythrocyte adhesion and invasion take place. They then multiplied in the infected erythrocytes, resulting in the formation of eight daughter cells. Infected erythrocytes can survive for weeks in the body [14].

*Bartonella* is the only genus known to produce factors that stimulate angiopoietin-2 production in endothelial cells and vascular endothelial growth factor production in epidermal cells. The BadA protein on the cell surface of *B. henselae* is responsible for adhesion to endothelial cells and fibronectin. In *B. henselae* infections, the BadA protein promotes angiogenesis. BadA protein has also been linked to angiogenesis via the inducible hypoxia factor-1 (IHF-1) pathway. The expression of outer membrane proteins (Vomp) is responsible for promoting vascular growth in *B. quintana*. Their ability to enter erythrocytes shields these species from the host’s adaptive and innate immune responses. CD4 T-helper cells produce interferon-gamma and TNF alpha, which are responsible for bacterial elimination. *Bartonella* species are capable of attenuating the host immune response, thereby establishing a chronic asymptomatic carrier state [14,15].

The diagnosis of BA is usually based on clinical features and histopathological evaluation. The characteristic histopathological features of BA include well-developed engorged capillaries with inflamed stroma. Numerous inflammatory cells with basophilic bacilli are noted. The clinical differential diagnosis includes Kaposi sarcoma and pyogenic granuloma.

Kaposi sarcoma is composed of ill-developed, slit-like spaces formed by atypical spindle cells [13], whereas pyogenic granuloma shows exuberant granulation tissue covered by hyperplastic or atrophic epithelium, which may show ulceration, numerous endothelium-lined blood vessels and proliferation of fibroblasts, and budding endothelial cells along with mixed inflammatory infiltrate [7]. So, based on the above histopathological findings, bacillary angiomatosis was given, which was also confirmed using special stains for bacterial colonies.

The Warthin-Starry stain is a silver nitrate-based stain for the detection of spirochetes and confirmation of *Bartonella henselae*. It stains the bacteria dark brown to black color, and the background appears light golden brown or golden yellow. The modified Brown and Brenn stain is differential staining for gram-positive and gram-negative bacteria. Gram-positive bacteria appear blue, and gram-negative bacteria and nuclei appear red with a yellow background. The present case showed black rod-shaped bacilli in clusters and superficial microbial colonies in the above stains [16].

Excellent responses were shown with the treatment with erythromycin, doxycycline, tetracycline, and azithromycin [17]. Lesions frequently relapse; hence, a periodic review is necessary to avoid complications.

## Conclusions

To avoid unnecessary complications during treatment, bacillary angiomatosis should be considered in the differential diagnosis of unclear masses and lymphadenopathy of the cervicofacial or orofacial region. On the basis of our findings, BA is one of the common elements in immunocompromised status such as HIV and diabetes, and a thorough history taking along with microbial and serological tests will make the way to a proper confirmatory diagnosis.
